# Lung Regeneration: Endogenous and Exogenous Stem Cell Mediated Therapeutic Approaches

**DOI:** 10.3390/ijms17010128

**Published:** 2016-01-19

**Authors:** Khondoker M. Akram, Neil Patel, Monica A. Spiteri, Nicholas R. Forsyth

**Affiliations:** 1 Academic Unit of Respiratory Medicine, University of Sheffield, Sheffield S10 2RX, UK; k.m.akram@sheffield.ac.uk; 2 Department of Respiratory Medicine, University Hospitals of North Midlands, Stoke-on-Trent ST4 6QG, UK; npuk@hotmail.co.uk (N.P.); Monica.Spiteri@uhns.nhs.uk (M.A.S.); 3 Guy Hilton Research Centre, Institute of Science and Technology in Medicine, Keele University, Stoke-on-Trent ST4 7QB, UK

**Keywords:** lung development, lung regeneration, lung stem cells, stem cell-mediated therapeutics, signalling pathways in lung repair, lung tissue engineering

## Abstract

The tissue turnover of unperturbed adult lung is remarkably slow. However, after injury or insult, a specialised group of facultative lung progenitors become activated to replenish damaged tissue through a reparative process called regeneration. Disruption in this process results in healing by fibrosis causing aberrant lung remodelling and organ dysfunction. Post-insult failure of regeneration leads to various incurable lung diseases including chronic obstructive pulmonary disease (COPD) and idiopathic pulmonary fibrosis. Therefore, identification of true endogenous lung progenitors/stem cells, and their regenerative pathway are crucial for next-generation therapeutic development. Recent studies provide exciting and novel insights into postnatal lung development and post-injury lung regeneration by native lung progenitors. Furthermore, exogenous application of bone marrow stem cells, embryonic stem cells and inducible pluripotent stem cells (iPSC) show evidences of their regenerative capacity in the repair of injured and diseased lungs. With the advent of modern tissue engineering techniques, whole lung regeneration in the lab using de-cellularised tissue scaffold and stem cells is now becoming reality. In this review, we will highlight the advancement of our understanding in lung regeneration and development of stem cell mediated therapeutic strategies in combating incurable lung diseases.

## 1. Introduction

The tissue turnover of unperturbed adult lung is remarkably slow. However, after injury or insult a specialised group of facultative lung progenitors become activated to replenish damaged tissue through a reparative process called regeneration. Interruption in this process results in healing by fibrosis causing scarring, aberrant lung remodelling and organ dysfunction [1,2]. A highly specialised group of endogenous lung stem/progenitor cells are believed to maintain lung homeostasis and regeneration. In this review we will discuss our latest understanding of the identity of lung stem cells, their activation and proliferation with subsequent differentiation to replenish or repair injured lung tissues. This will include a discussion of lung development and the associated signalling that governs this process. Evidence suggests that in some disease conditions, such as COPD, the embryonic lung developmental process could be reactivated [3,4,5]. Furthermore, for *de novo* derivation of lung progenitors from pluripotent embryonic stem cells (ESCs) and inducible pluripotent stem cells (iPSC) *in vitro*, it is essential to understand the molecular pathways that regulate differentiation of adult lung cells from a common embryonic foregut endoderm origin. Exogenous stem cells, such as mesenchymal stem cells (MSCs) and ESCs are involved in research designed to identify clinical applications in lung disease and this will also be investigated.

## 2. Lung Development and Associated Signalling Pathways

Mammalian lung development is a complex but continuous process that starts at approximately embryonic day 9 (E9.0) in mice, and at an undefined early time in human, and continues through infancy [6]. The early embryonic and pseudoglandular stages elaborate the conducting airways, and in later stage canalicular, saccular and alveolar development takes place through reduction of mesenchyme and formation of pulmonary vasculature to form a thin air-blood barrier. A new-born infant possesses approximately 50 million alveoli but will add another 250 million alveoli through infancy increasing surface area from approximately 3 to 70 m^2^. In a completely developed lung there are more than 40 different cell types, with different functions [7].

**Figure 1:**
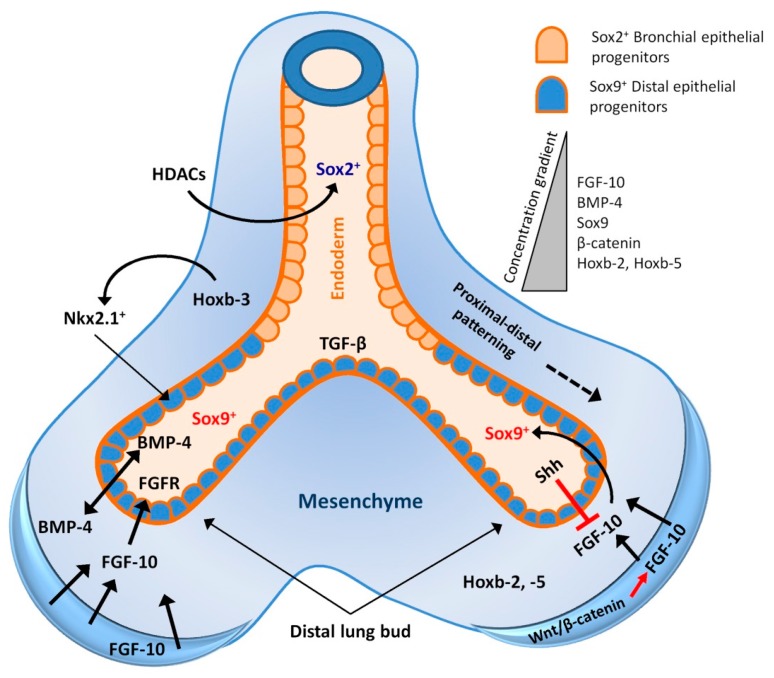
Epithelial-mesenchymal cross-talk and governing signalling during early development and branching morphogenesis of lung. Factors are represented only at sites where the expression is most abundant. Fibroblast growth factor-10 (FGF-10) is highly expressed in the distal mesenchyme and acts as a chemotactic focus for the epithelium during lung budding. FGF-10 also regulates Sox9 expression in the distal epithelial progenitors and induces bone morphogenetic protein (BMP)-4 expression. Sox2 expression in the proximal epithelium is under regulation of histone deacetylases 1/2 (HDAC1/2) signalling. FGF-10 expression in the mesenchyme is regulated by Wnt/β-catenin signalling (red arrow). A high concentration of BMP-4 signal also serves to locally inhibit endoderm proliferation, thereby inducing the lateral outgrowth of new airway branches. Sonic hedgehog (Shh) at the distal tips functions to downregulate FGF-10 expression in the mesenchyme, which limits local budding. Transforming growth factor-β (TGF-β) signalling also prevents local budding, by decreasing endodermal proliferation and by stimulating synthesis of matrix components at branch points. Solid arrows indicate sources from and influences on cells/molecules; dotted arrow indicates direction of patterning.

During embryonic weeks 3 to 7, the human lung originates as a ventral diverticulum arising from the foregut endoderm, which bifurcates and gives rise laterally to the two primary bronchial buds (Figure 1). The lung buds grow into surrounding splanchnic mesoderm and branch repeatedly, giving rise to the future respiratory tree. This primitive lung bud is lined by epithelium derived from foregut endoderm, which later differentiates into specialised cells that line both the conducting and respiratory airways [7,8]. On the other hand, mesenchymal cells condensed around the primitive airways differentiate into blood vessels, smooth muscle, cartilage, and other connective tissues of the lung [9]. During embryonic weeks 7 to 16, an extensive branching morphogenesis takes place through rapid proliferation of the primitive airways. This bronchial tree branching pattern does not change after this stage and remains more or less unchanged throughout the adult life [10]. This is the pseudoglandular stage. Here, the most peripheral structures including the terminal bronchioles, respiratory bronchioles and alveolar ducts form. The cartilage begins to form around the larger airways and smooth muscle forms around airways and major blood vessels. In this phase, the airways are lined by columnar epithelium with few ciliated and goblet cells [10,11].

During the canalicular and saccular stages (embryonic weeks 16 to 35), the pulmonary acinar units form which contain alveolar ducts, alveolar sacs and alveoli. A gradual decrease in mesenchymal tissue results in close apposition of the pulmonary vasculature to the alveolar walls. At this time, type I alveolar epithelial cell (AECI) and type II AEC (AECII) are differentiated from the cuboidal epithelial cells in the most peripheral parts of the lung. Lamellar bodies associated with surfactant synthesis begin to appear in the cytoplasm of AECII cells and AECI cells begin their flattening process and attenuate to provide an air-blood interface [11]. This alveolar development continues after birth and is completed through infancy [12].

During lung organogenesis, the proximodistal patterning, branching morphogenesis, alveolarisation and cellular differentiation are strictly regulated by the surrounding mesenchyme and a number of key growth factor-directed signalling pathways [13]. Cumulative data from several recombination experiments reveal that mesenchyme is essential for initial lung budding and subsequent branching of foregut endoderm, where the governing stimulation is carried out by the qualitative and quantitative differential distribution of surrounding mesenchyme [14,15,16,17]. This reciprocal endoderm-mesoderm cross-talk is likely mediated by numerus soluble growth factors and transcription factors originating from both epithelial and mesenchymal cells (Figure 1) [18,19,20]. Mouse embryonic lung organ culture experiments demonstrate that the quantity of mesenchyme specifies epithelial cell fate during differentiation. That is, a small amount of mesenchyme is able to direct cell differentiation toward a bronchiolar phenotype; whereas, an increased amount of the same mesenchyme is able to induce the epithelial cells to differentiate toward an alveolar phenotype [21]. Furthermore, tissue recombination experiments show that distal lung mesenchyme can reprogram rat tracheal epithelial cells to an AECII cell phenotype, and conversely, proximal mesenchyme can induce distal lung epithelium towards a tracheal epithelial cell phenotype [22,23]. These findings highlight the significance of the epithelial-mesenchymal interaction in coordinating the precise temporospatial pattern of lung development.

At least 11 different epithelial cell types have been identified in the conducting and respiratory portions of the lung [24]. The basal, secretory, and ciliated cells are the major cell types constituting the pseudostratified epithelium of the proximal tracheobronchial area while AECI and AECII cells make up the alveolar epithelium [25]. The onset of cell differentiation is signalled by various gene products including surfactant protein (SP)-A, SP-B, SP-C, SP-D and club (Clara) cells secretory protein (CCSP) [26,27,28,29]. During early embryonic development, the undifferentiated epithelium co-expresses several lineage markers including SP-A, CCSP and neuroendocrine cell markers. But later, during the pseudoglandular phase, this epithelium differentiates into different lineages and becomes restricted to proximal and distal airways [30]. Transcriptional analysis reveals that the surfactant proteins and CCSP genes share common mechanisms for gene regulation and expression in respiratory epithelial cells. Nkx2.1 (also known as thyroid transcription factor-1 (TTF-1)), a homeodomain protein, plays a pivotal role in several phases of lung development, including epithelial cell lineage determination [31,32,33,34]. Nkx2.1 binds and activates SP-A, SP-B, SP-C, and CCSP promoter elements as well as Nkx2.1 itself [35,36,37,38,39]. During development of the respiratory epithelium, Nkx2.1 appears to be the earliest marker, with onset of expression at the time of lung bud formation from the foregut endoderm [40]. Expression of Nkx2.1 is present throughout the foetal lung including respiratory epithelial cells of the trachea, bronchi, and developing respiratory tubules, but its expression is most robust in distal alveolar epithelium [24,40,41]. Null mutation of Nkx2.1 in mice exhibit tracheoesophageal fistulas and fail to form bronchiolar and alveolar structures [32,33,34]. Pulmonary-specific gene expression including *SP-B*, *SP-C*, and *CCSP* is extinguished within transgenic lungs, which do, however, contain ciliated and mucus-secreting cells [34]. Thus, Nkx2.1 is recognised as a “master gene” in maintaining the lung morphogenesis as well as cytodifferentiation of certain epithelial cell lineages [24]. However, targeted gene mutation studies confer that while Nkx2.1 is not required for initial specification of lung primordia it is essential for pulmonary development and cell differentiation [33,42].

The precise regulatory function of Nkx2.1 in pulmonary cytodifferentiation is not well understood; however, study reveals that Nkx2.1 has multiple binding sites for both ubiquitous and specific transcription factors, including those of the hepatocyte nuclear factor (HNF) and GATA zinc finger families [43,44,45]. GATA and HNF play crucial role for the development of the foregut endoderm [46,47,48]. Multiple studies have identified HNF-3 binding sites in the SP-A, SP-B, and CCSP promoter regions [35,49,50]. The HNF-3β null mutation results in an early embryonic lethal phenotype with primitive foregut deformities, resulting in agenesis of lung and other foregut derivatives [51]. While Nkx2.1, GATA and HNF play crucial role in cytodifferentiation and specification of cell fate, the Homeobox (*Hox*) genes regulate the patterning of the lung during embryonic development. *Hox* genes act as transcription factors and are consistently expressed throughout the lung during development and maintain proximal-distal orientation of the lung as well as branching morphogenesis [52,53,54]. *Hoxb-3* and *Hoxb-4* genes are expressed both in the proximal and distal mesenchyme of the entire developing lung; whereas, *Hoxb-2* and *Hoxb-5* are restricted within the mesenchyme of distal lung buds (Figure 1) [52]. Hoxb-3 transactivates the Nkx2.1 promoter, which suggests that Hoxb-3 could regulate proximal-distal lung patterning in an Nkx2.1 depended manner [24,31]. Mouse embryonic lung culture experimentation has demonstrated that retinoic acid induces *Hoxa-5*, *Hoxb-5* and *Hoxb-6* gene expression; whereas, Hoxb-5 is negatively regulated by epidermal growth factor (EGF) and transforming growth factor-β (TGF-β) [55,56]. Retinoic acid has been demonstrated to facilitate the growth of proximal airways and gene expression at the expense of distal structures in a dose-dependent manner; therefore, it is probable that *Hox* genes mediate the retinoic acid-induced alteration in lung patterning [57,58].

Bone morphogenetic protein (BMP)-4, a member of the TGF-β family proteins, is also implicated in the control of the proximal-distal patterning of the lung and in branching morphogenesis [58,59]. *BMP-4* gene expression is restricted to the tips of distal buds and to the adjacent mesenchyme, which locally inhibits endoderm proliferation and forces the outgrowth of lateral branches (Figure 1) [58]. Moreover, inhibition of BMP signalling results in complete proximalization of the respiratory epithelium, including ciliated cells in the most distal portions of lungs. Therefore, it is hypothesised that BMP proteins provide a concentration gradient to regulate proximal *versus* distal lung endoderm differentiation [59]. Endodermal cells located at the periphery of the lung, which are exposed to high levels of BMP-4, maintain a distal identity while cells below a certain threshold of the BMP-4 signal initiate a proximal differentiation program [24].

The Sox2 and Sox9 transcription factors mark lung bud endoderm as proximal and distal epithelial progenitors respectively (Figure 1). Sox2 regulates the differentiation of proximal progenitors into secretory and ciliated epithelium; whereas, Sox9 directs distal progenitors into alveolar epithelial cells [60,61,62,63,64,65]. During early lung development, fibroblast growth factor-10 (FGF-10), which is highly expressed in the distal mesenchyme and is regulated by Wnt signalling (Figure 1), acts on the distal lung epithelial progenitors to maintain them and prevent them from differentiating into proximal airway epithelial cells by inducing Sox9 and repressing Sox2 expression [66,67,68,69,70]. When the lung epithelium extends into the mesenchyme, more proximally located cells become less exposed to distally sourced FGF-10 and gradually start to differentiate [20,69,70,71,72]. In contrast, studies show that suppression of FGF-10 around the developing airway, as well as during late gestation and postnatal development, facilitates proper maturation of the lung epithelium [73,74,75,76]. Furthermore, ectopic overexpression of FGF-10 at later stages of lung development blocks the alveolar differentiation program via induction of Sox9 expression [66,72,77]. Therefore, FGF-10 is thought to maintain distal epithelial progenitor population helping branching morphogenesis during early lung development in a Sox9 dependent manner [78].

While Sox9^+^ progenitors are largely under regulation of FGF-10, the Sox2^+^ progenitors in the lung endoderm are regulated by histone deacetylases 1 (HDAC1) and HDAC2 (Figure 1). HDAC1/2 deficiency leads to a loss of Sox2 expression resulting in suppression of proximal airway development. This is mediated in part by de-repression of BMP-4, which is direct transcriptional target of HDAC1/2. In contrast to development, postnatal loss of HDAC1/2 in airway epithelium does not affect the expression of Sox2 or BMP-4 [79].

Despite high-sequence homology and affinity for the same cognate receptor, FGF-7 and FGF-10 exert distinct effects on the developing lung. FGF-7 predominantly influences airway branching by promoting epithelial cell proliferation and expansion [18,80,81]. FGF-7 acts as differentiation factor in developing lung. Lung organotypic culture experiments show that in absence of any mesenchyme or serum, exogenous application of FGF-7 can induce AECII-like epithelial cell differentiation [81,82]. During postnatal life, alveolarisation is regulated by cooperative interaction of various FGFs with FGFR-3 and FGFR-4 receptors [83]. Double mutation of these receptors results in postnatal pulmonary lethality where alveolarisation is severely disrupted leading to an emphysematous phenotype. This transgenic mouse phenotype also exhibits an abnormal elastin synthesis causing a lethal form of parenchymal defect [83]. Furthermore, inactivation of the epidermal growth factor (EGF) receptors affects AECII cell maturity, with decreased expression of the SP-C protein. In contrast, exogenous EGF administration accelerates alveolar type-II cell differentiation in foetal lungs [84].

## 3. Endogenous Stem Cell Population of the Respiratory System

**Figure 2:**
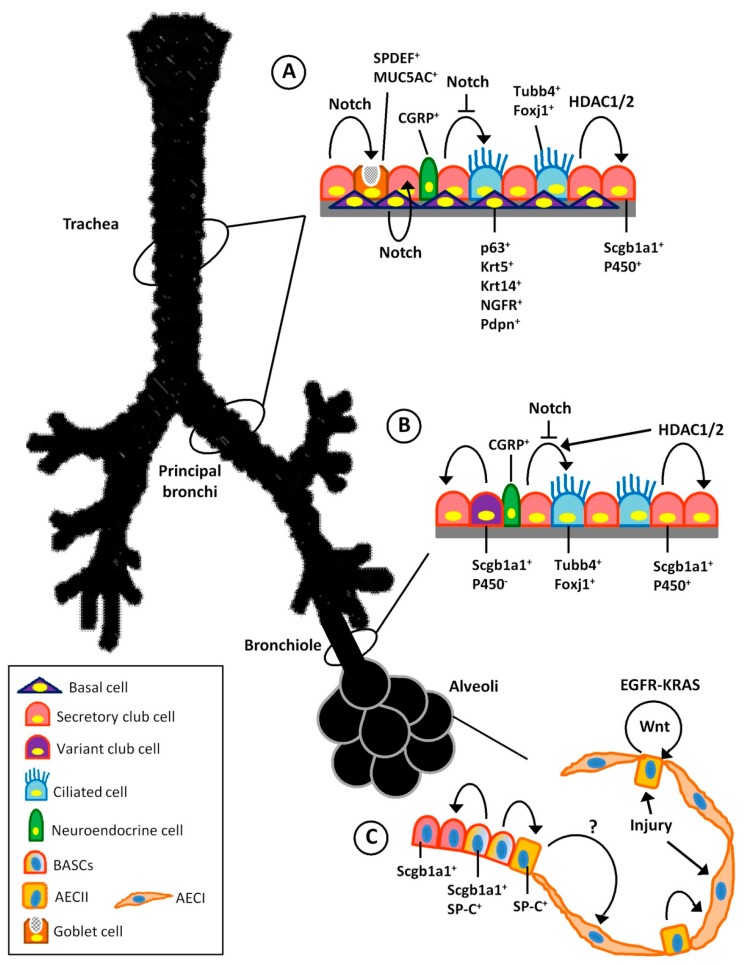
Endogenous stem cells in the airways and alveoli with their differentiation in the postnatal lung. (**A**) The trachea and bronchi of the rodent and human lung are lined with multiple epithelial lineages. Basal cells are located in this region and can generate secretory club and ciliated cell lineages. Notch signalling is crucial for differentiation of basal cells and also suppresses ciliated cell differentiation. HDAC1 and HDAC2 are essential for secretory epithelial regeneration; (**B**) The bronchiolar lining epithelium of rodents lacks basal cells but contains variant club cells, secretory cells and ciliated cells; (**C**) Progenitor cell populations and their differentiated progeny in the lung alveolus. The alveolar epithelium consists of alveolar epithelial cell AECI and AECII cells. AECII cells can generate AECI cells during homeostasis and after injury. Generation of alveolar epithelium by other cells, such as BASCs has yet to be supported by lineage tracing. The AECII to AECI differentiation signalling is not clear (marked by “?”), but AECII self-renewal and proliferation occurs via EGFR/KRAS signalling. Curved arrows indicate differentiation and line arrows indicate molecular or physical stimulus.

Endogenous adult lung stem and progenitor cells are regenerative cell populations important for epithelial cell homeostasis and injury repair. In multiple adult organs, tissue-specific stem cells have been identified as multipotent cells with the capacity for long-term self-renewal and the ability to differentiate into other cell lineages. Tissue-specific stem cells are typically quiescent in normal conditions and proliferate during injury repair [85,86,87]. The unperturbed adult lung is a remarkably quiescent. Therefore, the detection of proliferative postnatal stem or progenitor cells that actively take part in repair and regeneration has been challenging. Numerous studies attempting to identify adult stem or progenitor candidates in the lung have used injury and disease models, primarily in rodents. To date, studies of human and animal lung morphology have identified subsets of lung epithelial cells with self-renewal and differentiation capacity, including basal cells (BC), secretory club cells (formerly known as Clara cells) of the proximal and distal airways, and AECII cells of the alveoli (Figure 2) [6]. All of these cells have the capacity to enter the cell cycle in response to lung injury [88,89,90,91,92,93,94,95]. More recently, advanced lineage tracing techniques have suggested that most types of lung epithelial cells, except for ciliated cells, can proliferate and expand after injury to promote repair [96,97,98,99,100]. Identifying these stem/progenitor cell populations and understanding their healing mechanism is important to harness the endogenous abilities of the lung to regenerate from a therapeutic perspective.

### 3.1. Trachea and Proximal Airways Stem Cells

The trachea and the principal bronchi (proximal airways) of both humans and mice are lined with a pseudostratified epithelium composed of basal and luminal cells. Luminal airway cells include secretory club cells, ciliated cells and neuroendocrine cells. In the humans and other mammals, the airway epithelium also contains SPDEF and MUC5AC expressing goblet cells. In this region, secretory club cells are marked by expression of secretoglobin family 1a member 1 (Scgb1a1^+^) [6]. The basal cells, on the other hand, are believed to be the most important stem cells of upper airways, marked by the expression of basal cell-restricted transcription factor p63, keratin (Krt)-5 (Krt5), Krt14, nerve growth factor receptor (NFGR) and cell-surface marker Pdpn (Figure 2A) [101,102,103]. This basal cell population, both in mice and human, have extensive proliferative potential, self-renewal capacity and ability to differentiate into secretory club and ciliated cells [102,104]. The 3D “tracheosphere” culture of FACS-sorted pure population of Krt5^+^p63^+^NGFR^+^ basal cells shows clonal expansion of differentiated cells which are positive for both ciliated and secretory club (Clara) cells [102,103].

An H1N1 influenza virus-induced mouse lung injury model recently demonstrated that following a sub-lethal viral infection p63^+^ basal cells in the bronchiolar epithelium underwent rapid proliferation and migration to damaged alveolar regions. Then, these migrated cells assemble into discrete, Krt5^+^ pods and express typical alveoli-associated markers. This study suggests that this p63^+^Krt5^+^ cell population plays a role as an intermediate in the reconstitution of the alveolar-capillary network eradicated by viral infection [105]. On the other hand, lineage-tracing studies in mice show that in an injury condition Scgb1a1^+^ secretory club cells can give birth to goblet cells, but goblets cannot be derived from ciliated cells [106]. Therefore, these basal and club cell population are considered as major stem/progenitor cells in the upper airways. The molecular mechanisms for basal cell self-renewal and differentiation are poorly understood. However, mouse tracheobronchial epithelium shows that Notch signalling is greatly enhanced during repair and is required for activation in basal cells promoting their luminal differentiation, primarily toward secretory lineages (Figure 2A). Signalling is also conserved in basal cells of the human airway [107].

### 3.2. Distal Airway Stem Cells

The distal bronchiolar region of mouse lungs are lined with a single-layered epithelium consisting of secretory club cells (Scgb1a1^+^ and Scgb3a2^+^), ciliated cells (Foxj1^+^ and Tubb4^+^) and neuroendocrine (NE) epithelial cells (CGRP^+^) (Figure 2B). In human, an additional population of Krt5^+^p63^+^ basal cells are also present in the bronchiolar lining of the airways, but their presence has not been well documented in the rodent lung [108]. Neuroendocrine cells are clustered within the epithelium as neuroendocrine bodies (NEBs) and are marked by expression of calcitonin gene-related peptide (CGRP). Lineage tracing studies show that CGRP^+^ NE cells are capable of self-renew in uninjured adult lung and can differentiate into secretory club and ciliated cells after naphthalene injury [109].

The unperturbed bronchiolar epithelium is remarkably quiescent. However, depletion of bronchiolar epithelium by naphthalene-induced injury spares a subset of secretory club cells that express Scgb1a1 but not cytochrome p450. These Scgb1a1^+^p450^−^ club cells, which survive from naphthalene toxicity, are designated as facultative progenitors; and are capable of expanding rapidly to regenerate the damaged airways by differentiating into new secretory club and ciliated cell populations (Figure 2B) [108,110]. Moreover, Scgb1a1^+^p450^−^ club cell populations also can self-renew and differentiate into ciliated epithelium during normal homeostatic turnover [111]. This particular subset of naphthalene resistant club cells is often called “variant club cells”. Interestingly, these variant club cells are mostly localised surrounding NEBs and at the bronchoalveolar duct junctions (BADJs) [110,112]. Activity of Notch signalling in the NEB microenvironment has recently been identified [113,114]. Parabronchial smooth muscle cells, which line most of the proximal airways, were proposed to help make up a niche capable of activating variant club cells. It has been shown that naphthalene injury stimulates FGF-10 secretion by the parabronchial smooth muscle, which appears to be necessary for regeneration of variant club cells [115].

The degree of injury has differential effects in its modulation of secretory club cell mediated airway regeneration. For instance, mild injury initiates proliferation and expansion of randomly distributed club cells, which in turn differentiate into ciliated epithelium and thus promote repair. On the other hand, severe naphthalene injury results in activation of variant club cells located around NEBs and within the BADJ which then regenerate the injured tissues [116]. A growing body of literature supports the notion that region-specific variant club cells of the NEB and BADJ niches preferentially contribute to bronchiolar repair and regeneration after significant depletion of secretory cells, most likely via Notch and FGF-10 regulation [6].

### 3.3. Stem Cells of the Alveolar Region

Pulmonary gas exchange occurs in alveolar sacs which are lined by squamous type AECI and cuboidal AECII cells. Squamous AECI cells are terminally differentiated cells that line approximately 95% of the alveolar surface through which gas exchange takes place; whereas, AECII cells secret surfactant proteins that prevent alveolar collapse [117]. Classically, AECII cells are categorised as the progenitors for AECI cells which replenish alveolar lining during normal tissue turnover and during repair after injury (Figure 2C) [118]. Significant numbers of *in vivo* studies have demonstrated that postnatal injury to alveoli initiates AECII cell proliferation and differentiation into AECI cells in the attempt of alveolar repair [93,94,119,120,121,122,123]. Recent genetic lineage-tracing experiments, combined with *in vitro* experiments, showed that SP-C positive AECII cells self-renew and differentiate into type I AEC progeny over about a year in normal conditions, however, if many AECII cells are specifically ablated, the individual survivor AECIIs undergo rapid clonal expansion and daughter cell dispersal. In addition, when Individual lineage-labelled AECIIs are placed into 3D culture, those cells give rise to self-renewing “alveolospheres”, containing cells which express both AECII and AECI specific markers [124].

Lineage tracing and clonal analysis have demonstrated that during development, AECI and AECII cells arise directly from a bipotent progenitor cell; whereas, after birth new AECI cells derive from a rare subtype of, self-renewing, long-lived, mature AECII cell that produces slowly expanding clonal foci of alveolar renewal. This stem-cell like AECII function is broadly activated by hyperoxic AECI injury. Furthermore, purified EGFR ligands, such as TGF-α, heparin-binding EGF, and NRG1 stimulate AECII proliferation *in vitro*. However, none of these EGFR ligands can induce AECII differentiation into AECI cells. On the other hand, oncogenic KRAS has shown to stimulate multifocal clonal AECII cell expansion *in vivo* [125]. Thus, it has been postulated that alveolar injury induces a signalling pathway transduced by EGFR-KRAS that controls AECII self-renewal, whereas a different signal (as yet unknown) controls AECII differentiation into AECI cell type (Figure 2C) [125].

In the mouse, another population of cells which co-express Scgb1a1 and SP-C markers are found at the BADJ area designated as bronchioalveolar stem cells (BASCs) (Figure 2C). These Scgb1a1^+^Sp-C^+^ BASCs can expand *in vivo* after bleomycin-induced lung injury, and the isolated cells can be clonally expanded *in vitro*, where they show multipotent differentiation into bronchiolar and alveolar lineages [126]. Lineage-tracing studies demonstrate that during development or after hyperoxic alveolar injury, the BASCs do not contribute in alveolar reconstitution [71]. In contrast, bleomycin-induced lung injury model shows that the BASCs are capable of generating AECIIs, although their contribution is limited, and they do not seem to expand substantially [127,128]. Furthermore, co-expression of Scgb1a1 and SP-C is not unique to BASCs, occasionally some AECIIs found throughout mouse alveolar regions also co-express both Scgb1a1 and SP-C [127]. As Scgb1a1 lineage tracing does not entirely distinguish airway BASCs from rare AECII cells, further confirmation is required before defining BASCs as alveolar stem cells [6].

### 3.4. Lung Mesenchymal Stem Cells

Mesenchymal stem cells (MSCs) are present in the stromal tissue of many organs, including bone marrow, adipose tissue, muscle, periosteum, dermis, and lung [129]. A multitude of studies have confirmed the presence of MSCs in the lungs of mice [130,131,132,133,134], humans [135,136,137], and sheep [138,139]. Lung-derived MSCs share some common features with conventional bone marrow-derived MSCs and fulfil the International Society of Cellular Therapy criteria for MSCs, including marker profile and differentiation capacity [140]. Lung MSCs are also clonogenic when grown on Matrigel with bFGF or hepatocyte growth factor [141,142]. However, the function of these lung MSCs is not completely understood.

Lung MSCs localised at the distal tip of the branching airway epithelium secrete FGF-10, a critical component of the signalling network that regulates BMP, Wnt, and sonic hedgehog (Shh) pathways necessary for cytodifferentiation in the developing lung [143]. Lung MSCs also support the proliferation and differentiation of airway epithelial stem cells in co-culture [144]. Recently, Hoffman and colleagues showed that intravenous administration of lung MSCs alleviates elastase-induced emphysema and increases survival through a paracrine-mediated anti-inflammatory mechanism [145].

## 4. Signalling Pathways Involved in Postnatal Lung Injury Repair and Regeneration

To understand the molecular mechanisms that regulate the activation, expansion, and differentiation of stem and progenitor cells in response to injury and repair is crucial to aid development of targeted therapeutic strategies to combat lung diseases. The surrounding mesenchyme also plays critical role in promoting lung stem cells during injury repair [146]. Unilateral pneumonectomy (PNX) demonstrated that resection of a single lobe, leaving other lobes, intact, resulted in regrowth of alveoli within the remaining lungs [147,148,149]. This “neoalveolarisation” occurs through activation and proliferation of bronchiolar cells, AECII cells, endothelial cells and PDGFRα^+^ stromal cells [150,151,152,153,154,155]. The post-PNX progenitor cell activation and alveolar regeneration is believed to be regulated by elastin content, and EGF/FGF mediated signalling pathways [156,157]. Other signalling pathways, including Wnt and Notch, are known to play important roles in stem cell self-renewal and differentiation and to play key roles in lung repair and regeneration.

### 4.1. Wnt Signalling

Wnt signalling is an essential regulator of early lung endoderm specification and development and has been implicated in regulating regenerative responses [146]. During postnatal lung repair and regeneration canonical Wnt signalling is activated in the epithelium of various lung compartments [158,159,160,161,162]. Naphthalene-induced selective depletion of secretory club cells of airway epithelium activates Wnt signalling. This activation of Wnt signalling through loss of the transcription factor GATA6 leads to expansion of the BASCs population after naphthalene injury [163]. However, canonical Wnt signalling may not be essential for lung regeneration as postnatal deletion of β-catenin does not obliterate secretory cell proliferation [163]. Moreover, loss of β-catenin in postnatal alveolar epithelium leads to increased fibrosis and AEC death [164]. In addition, blocking of Wnt signalling by pharmacologically active molecules can reduce bleomycin-induced lung fibrosis [165]. Furthermore, increased activation of Wnt signalling has been associated with idiopathic pulmonary fibrosis (IPF) lesions [166]. Therefore, precaution should be taken with the manipulation of Wnt signalling in the activation of lung progenitors to promote lung regeneration. Excessive activation of Wnt signalling may lead to fibrosis and other deleterious effects [6].

### 4.2. Notch Signalling

Notch signalling plays an important role in airway epithelial regeneration. During lung development, Notch signalling is essential for differentiation of the secretory epithelium from proximal lung endoderm [167,168]. In postnatal lung injury, Notch signalling is essential for differentiation of basal cells into secretory cells [107]. Increased Notch activation can expand the secretory lineage at the expense of the ciliated lineage [107,169]. In addition, reactive oxygen species (ROS) activate Notch signalling via Nrf2 activation. The ROS-Notch pathway is important for BC self-renewal through regulation of cell proliferation, which may be critical for maintaining the BC population in the upper airways of rodent and human lungs [170]. This unique ability of Notch to promote a specific secretory cell lineage differentiation may offer a potential therapeutic target to activate lung resident stem cells towards promotion of airway epithelial regeneration after acute lung injury [6].

### 4.3. Histone Deacetylases (HDACs) Signalling

Histone deacetylases (HDACs) are enzymes that eliminate acetyl groups from histones and modulate gene function. Genetic deletion of HDAC1 and HDAC2 in postnatal secretory club cells in mice led to the induction of tumour suppressor genes Rb1, p21, and p16 after naphthalene injury, which resulted in inhibition of club cell proliferation and permanently impaired epithelial regeneration [79]. This finding indicates that HDAC1 and HDAC2 may play important role in airway epithelial regeneration after injury in postnatal lung. HDAC may also play important role in the regulation of AECII function during development and regeneration in a Hop-dependent manner. Hop is an unusual homeodomain protein expressed in airway epithelium coincident with HDAC2, acting downstream of Nkx2.1 and GATA6 signalling pathways to negatively regulate surfactant protein expression by the AECII cells. Loss of Hop expression *in vivo* results in defective AECII cell development with increased surfactant production and disrupted alveolar formation. This Hop-mediated regulation of alveolar maturation is found to be HDAC-dependent [171]. Individuals with COPD have a significant decrease in HDAC activity in their lung epithelia with an associated complete loss of HDAC2 expression [172]. In asthma, bronchial epithelium displays reduction of HDAC activity as well [173]. Taken together, this suggests that HDACs have an important role in pulmonary epithelium homeostasis and repair. Therefore, pharmacological interventions targeting HDACs to increase histone acetylation and gene expression could have therapeutic benefits in promoting lung regeneration.

### 4.4. miRNAs and lncRNAs Regulation

Noncoding micro RNAs (miRNAs) and long noncoding RNAs (lncRNAs) play crucial roles in epithelial branching and differentiation during lung development. Two miRNA clusters, miR17-92 and miR302-367 play important roles in lung epithelial proliferation and differentiation. Both of these clusters are highly expressed during early lung development and then are significantly downregulated by birth [174,175]. Overexpression of these miRNA clusters results in increased lung epithelial proliferation at the expense of differentiation inhibition [174,176]. On the other hand, a recent study identified hundreds of lncRNAs expressed in the developing and postnatal lung [177]. A subset of these lncRNAs is located near to transcription factors Nkx2.1 and Foxf1. One of these lncRNAs, called NANCI, has been found to regulate SP-C and Scgb1a1 expression. Therefore, modulation of noncoding RNA candidates could be another way to activate and pulmonary regeneration process during disease condition as therapeutics [146].

## 5. Effects of Matrix Components in Lung Regeneration

The focus of this review is primarily to discuss the cellular components and their participation in lung regeneration; however extracellular matrix (ECM) also plays an important role. The ECM components play key roles in lung development, injury repair, regeneration and disease pathogenesis [178]. The major components of the lung ECM are collagen and elastin, interwoven with fibronectin and proteoglycans, that provide plasticity of the lung parenchyma [179]. Other important native lung ECM components are laminins, heparan sulfate proteoglycans, entactin, hyaluronate, chondroitin sulfate, and glycosaminoglycans [180]. In the lung, collagen subtypes I and III provide structural integrity, while collagen IV is predominantly a basement component [180,181]. Fibronectin is important for the adherence of a variety of pulmonary cell types to the ECM, and regulates cellular morphology, motility, and differentiation [182]. A wide range of matrix molecules mediate the fate of epithelial cell differentiation. For instance, AECIIs maintain their phenotype on collagen and laminin-5 substrates, but differentiate towards AECIs when cultured on collagen or laminin-5 alone [183,184]. ECM also significantly impacts stem cell differentiation. It has been reported that laminin-332 and Matrigel coatings led to increased SP-C expression by ESC-derived AECII cells [185]. For more understanding in the effects of ECM in the stem cell niche, physical stimuli and ECM-growth factor interaction in lung regeneration the following ECM-focused reviews are recommended [186,187].

## 6. Exogenous Stem Cell Interventions in Regenerative Therapies

### 6.1. Differentiation of ESCs and iPSCs into Pulmonary Epithelium

Pluripotent embryonic stem cells (ESCs) and induced pluripotent stem cells (iPSCs) offer enormous potential for regeneration of injured tissue and repair of disease states. ESCs are isolated from the inner cell mass of preimplantation blastocysts of an early mammalian embryo, and they can be maintained indefinitely in an undifferentiated state with the ability to differentiate into cells of all three embryonic germ layers [188]. On the other hand, iPSCs are classically generated by reprogramming fibroblasts by retroviral transduction of the transcription factors Klf4, Sox2, Oct4, and c-Myc. Once generated, iPSCs show all features of pluripotent ESCs and are capable of differentiation into cells of all three embryonic germ layers [189,190,191]. The possibility of patient-specific iPSCs offers great hope for generating genetically matched patient-specific lung progenitor cells, with the opportunity for patient specific regenerative cell therapy and drug screening as well as *in vitro* modelling of human diseases [87].

ESCs, in *in vitro* culture can perform differentiation reminiscent of the early embryo; therefore, efforts have been taken to direct differentiation of ESCs towards lung progenitors by applying signalling transduction that occurs during lung development. Definitive endoderm progenitor cells of the developing foregut give rise to the differentiated tissue of the thyroid, lung, liver, and pancreas. The lung epithelia development progresses through a primordial progenitor stage defined by the onset of Nkx2.1 expression and downregulation of Sox2 [34,40,63]. In the later stage of development, Sox2 expression increases in the area of the future lung trachea, bronchus, and bronchioles where the Nkx2.1-expressing progenitor cells of the embryonic lung giving rise to mature airway epithelium [63]. Sox9 and FoxP2 are expressed in the distal embryonic lung and mark a multipotent embryonic lung progenitor population [192,193].

Initial attempts to generate functional lung epithelium from ESCs generated mixed cell populations with the risk of teratoma formation after transplantation because of the remaining undifferentiated pluripotent stem cells [194]. To overcome this risk, Green *et al.* [195] found that dual inhibition of TGF-β and BMP signalling after specification of definitive endoderm (directed via Activin treatment) from ESCs highly enriches anterior foregut endoderm for the next steps in differentiation. Recently, definitive endoderm has been derived from mouse ESCs and converted first into foregut endoderm, and then into Nkx2.1^+^ lung endoderm using precisely-timed TGF-β, BMP-4, FGF-2, and Wnt signalling. Nkx2.1^+^ lung endoderm was then converted into multipotent embryonic lung progenitor and airway progenitor cells with the formation of “tracheospheres” when subcutaneously transplanted into nude mice [196]. Using the same differentiation technique, Mou *et al* [196] were able to generate disease-specific lung progenitor cells from human Cystic Fibrosis iPSCs, and these lung progenitors formed respiratory epithelium when subcutaneously engrafted into immunodeficient mice.

Similarly, Longmire *et al.* [197] purified and directed differentiation of ESCs following initial inhibition of TGF-β and BMP signalling, and then via stimulation of BMP-4 and FGF-2 signalling to form definitive endodermal precursors able to re-cellularise a 3D lung scaffold. An *in vitro* directed differentiation protocol has also been developed for generating functional cystic fibrosis transmembrane conductance regulator (CFTR)-expressing airway epithelia from human ESCs [198]. Carefully timed treatment by exogenous growth factors (FGF-7, FGF-10, FGF-18 and BMP-4) followed by air-liquid interface culture stimulated the differentiation of hESCs into airway epithelial cells that express functional CFTR [198]. Very recently, Huang and colleagues have derived lung progenitor cells (with ~80% efficiency) from hESC/iPSCs, which were capable of differentiating into airway and alveolar epithelial cells *in vitro* and *in vivo* [199]. Through refinement of the differentiation protocol, they first derived definitive endoderm from hESC (and iPSC) by Activin/BMP-4/bFGF treatment. This definitive endoderm was then sequentially exposed to the BMP inhibitor dorsomorphin, TGF-β inhibitor SB431542 and endogenous Wnt blocker IWP2 for shorter period, followed by a longer exposure to CHIR99021 (a small molecule that mimics Wnt signalling), FGF-10, KGF, BMP-4 and retinoic acid for derivation of anterior foregut endoderm. These lung progenitors were then differentiated into variety of pulmonary epithelial/progenitor cells including basal cells, club (Clara) cells, goblet cells, ciliated cells, AECII and AECI *in vivo* in immunodeficient mice and *in vitro* culture [199]. Tremendous efforts to better understand the details of ESCs- and iPSCs-directed differentiation in the pulmonary field are ongoing, showing great progress and therapeutic promise.

### 6.2. MSC-Mediated Regenerative Therapies

MSCs have been recently evaluated as a potential cell-based therapy for lung diseases. Preclinical studies and clinical trials demonstrate that the application of MSC stimulates wound repair and regeneration with efficient amelioration of a number of clinical conditions [200,201,202,203]. The precise mechanism of MSC-mediated wound repair and regeneration is not clear; however, evidence suggests that paracrine mechanisms are likely to be involved. One of the unique properties of MSCs is their site-specific migration and engraftment to injured tissues and differentiation into specific cell types. A variety of experimental animal models suggest active participation in wound repair and tissue regeneration [201,202,203]. On the other hand, some studies demonstrate that MSC-secreted paracrine factors play a vital role for wound repair, most likely through their anti-inflammatory, anti-apoptotic, angiogenic and immunomodulatory properties [204,205,206,207,208,209]. Additional reports suggest that MSC secretory products are capable of stimulating tissue-specific regional progenitor cells propagating tissue regeneration [210,211]. Recent evidence suggests that in addition to releasing soluble anti-inflammatory factors, the MSCs transfer microvesicles containing mitochondria, protein, and microRNAs to other cells [212,213]. Paracrine-modulating factors secreted by MSCs appear to be responsible for injury repair rather than MSC engraftment [213]. MSCs can be readily isolated from a variety of human tissues including bone marrow, adipose tissue, placenta, Umbilical cord and peripheral blood [214]. MSC-based regenerative therapy has been studied in pre-clinical animal models of several incurable and devastating lung diseases, with promising results are outlined below (reviewed in [214]).

Acute respiratory distress syndrome (ARDS) is a common and devastating clinical syndrome of acute lung injury (ALI) caused by various direct and indirect insults including infection, trauma, and major surgery. It can result in respiratory failure and ultimately death [215]. Several animal model studies demonstrate compelling data on the beneficial effects of MSCs in resolving acute lung injuries induced by endotoxin [216,217,218,219], hyperoxia [220], pneumonia [221] and systemic sepsis [206]. In a recent description, endotoxin-induced lung injury in explanted human lungs was ameliorated with the infusion of MSCs [206]. The accumulation of this pre-clinical data offers considerable hope that MSCs could be a potential candidate for the effective therapy of ARDS. Very recently, a Phase I clinical trial has been completed to evaluate the safety of single dose intravenous administration of allogenic bone marrow-derived MSCs in a cohort of nine moderate-to-severe ARDS patients. Single dose MSCs administration was well tolerated without any intervention-associated adverse effect or death [222]. Based on this result, a Phase II clinical trial has been started.

Evaluation of the lipopolysaccharide (LPS)-induced mouse ALI model demonstrates that intravenous or intratracheal administration of MSCs significantly attenuated LPS-induced pulmonary inflammation and alveolar injuries, improved alveolar fluid clearance, and reduced mortality [217]. This improvement of the pulmonary condition occurs in the absence of any significant engraftment of MSCs in the lung, suggesting a paracrine role of MSCs in the alleviation of ALI via suppression of pro-inflammatory cytokines tumour necrosis factor (TNF)-α and upregulation of anti-inflammatory cytokine IL-10 [217]. In support of MSC-paracrine mediated anti-inflammatory effects, Ortiz and colleagues demonstrates that MSCs and/or acellular conditioned media collected from cultured MSCs attenuates acute pulmonary inflammation. This attenuation was via suppression of both IL-1α-dependent T-lymphocyte proliferation and inhibition of TNF-α secretion by activated macrophages via MSC-secreted IL-1 receptor antagonist *in vitro* and in the bleomycin-induced murine lung injury model [207]. Furthermore, under inflammatory condition MSCs can be stimulated by pro-inflammatory cytokines and endotoxins, such as TNF-α and LPS. Endotoxin-mediated MSC activation occurs via toll-like receptor-4, resulting in increased production of cyclooxygenase-2 and increased prostaglandin-E2 release. MSC-secreted prostaglandin-E2 leads increased macrophage IL-10 secretion and thus can attenuate sepsis and sepsis-associated lung injury [206].

The therapeutic potential of MSCs has also been tested and shows promise in several chronic lung conditions including chronic obstructive pulmonary disease (COPD), idiopathic pulmonary fibrosis (IPF), bronchial asthma and pulmonary hypertension (PH). PH is a progressive disease with ongoing endothelial dysfunction and vascular remodelling. MSC administration has shown therapeutic benefits in murine models of PH [213]. Moreover, genetically engineered MSCs that overexpress endothelial nitric oxide synthase, prostacyclin, or heme oxygenase-1 had even greater reversal effects on PH [223,224].

IPF is a progressive disease currently without any effective therapy [225,226,227]. MSC delivery to the bleomycin injury IPF mouse model has shown low levels of MSC engraftment, but significant improvement in lung injury and fibrosis [228,229,230]. In 2007, Ortiz and colleagues showed that MSCs protects against bleomycin-induced lung injury and reduced fibrosis by blocking pro-inflammatory cytokines such as TNF-α and IL-1 by MSC-associated IL-1 receptor antagonist [207]. The administration of KGF-expressing MSCs in the bleomycin-induced mouse lung fibrosis model has shown reduced fibrosis via suppression of collagen accumulation [228]. KGF has an established role in the repair of alveolar epithelium through stimulation of AECII cell proliferation, migration and spreading [231,232,233]. Ongoing work continues to explore the possible benefits of treatment with MSCs for this lung disease with high morbidity and mortality.

COPD is the fourth leading cause of death worldwide and has been projected to be the third leading cause in 2020 [234]. No curative therapy is available for COPD at this time. COPD is characterised by an ongoing cycle of repeated destruction and repair of bronchilo-alveolar regions with subsequent tissue remodelling and sustained irreversible airway obstruction [235]. Systemic administration of bone marrow-derived MSCs was shown to ameliorate the emphysematous changes in the irradiation and papain-induced experimental mouse COPD models [208]. In this model, transplanted MSCs were localised to the emphysematous lung parenchyma and had differentiated into AECII cells. This was accompanied by reduced alveolar epithelial cell apoptosis, via Bcl-2 expression, and reduced enlargement of alveolar spaces [208]. Autologous intratracheal transplantation of bone marrow MSCs significantly mitigated elastase-induced COPD in the rabbit model [236]. The transplantation of bone marrow stem cells was associated with improved lung function, an attenuation of inflammation, an inhibition of epithelial apoptosis, a decrease in matrix metalloproteinase-2 expression, and the stimulation of alveolar and bronchiolar cell proliferation where engraftment and differentiation of the transplanted stem was negligible [236].

A recent placebo-controlled, randomised, multi-centre clinical trial has examined treatment with systemic MSCs for COPD and showed neither any adverse effects nor any differences in pulmonary function tests or quality of life indicators [237]. This clinical trial showed important safety data, and it provides a basis for further clinical trials to test the efficacy of MSC treatment in respiratory diseases. Extensive work is ongoing to investigate the role of treatment with MSCs in lung diseases in both mouse models of respiratory disease and human clinical trials.

## 7. Stem Cells in Lung Tissue Engineering

Research in the tissue engineering field is focused on exploration of 3-dimensional tissue culture systems for use in development of functional lung tissue. The ultimate ambition of these studies is to reduce donor-dependent lung transplantation [238,239]. The unique architecture of the lung and its anatomical and physiological complexity presents a major challenge to tissue engineers. Tissue engineered tracheas have been developed using MSCs isolated from various sources before being cultured on biodegradable and biosynthetic scaffolds to generate tracheal cartilage for the repair of congenital tracheal defects in both animal and human clinical trial models [240,241]. Very recently, a group of tissue engineers, stem cell researchers, and clinicians developed a functional human airway by culturing MSC-derived chondrocytes on an acellular tracheal scaffold, which was subsequently transplanted in a female patient who had suffered airway damage from tuberculosis [242]. A de-cellularised 7-cm long segment of human trachea was taken from a 51-year-old white female donor and then the recipient’s bone marrow-derived MSCs were differentiated into chondrocytes and airway epithelial cells were cultured *in vitro*. The MSC-derived chondrocytes were seeded on the external surface of the de-cellularised trachea and airway epithelial cells seeded on the luminal surface, in an equal ratio, and cultured in an air-liquid interface rotating bioreactor for 96 h. After *in vitro* preparation, the tracheal construct was transplanted to the left bronchus of the recipient, which improved breathing difficulties without graft rejection [242]. This achievement encourages the development of more complicated parts of the lung, such the alveoli and pulmonary vasculature, and even the entire lung.

More recently, whole-lung tissue engineering has been attempted in animal models [243,244]. These approaches make use of whole-lung de-cellularisation strategies [245] followed by reseeding the de-cellularised lung scaffold with primary airway epithelial cells, vascular endothelial cells [243,244], whole lung cell suspension digests [243], MSCs [246,247], or differentiated ESCs or iPSCs [197,248,249]. After culture within a bioreactor, the organs were re-implanted into syngeneic recipients and demonstrated efficient gas exchange [243,244]. The strategies for de-cellularising whole lungs followed by re-cellularisation with lung progenitors/stem cells have been applied to pigs, nonhuman primates [250], and human [251] lungs [252,253,254]. However, the complexity of human lung structure and difficulties in reseeding cells into all desired compartments of lung have made this task challenging and will take many years to establish and transplant a fully functional tissue-engineered whole-lung for clinical use.

## 8. Conclusions

Lung disease is a major cause of morbidity and mortality worldwide. Despite being highly quiescent, the lung’s ability to regenerate extensively after injury suggests that this regenerative capability could be promoted and utilised in disease condition where loss of lung tissue occurs, and the lung fails to regenerate. Endogenous stem cells are indispensable during normal tissue turnover and repair or regeneration after injury to restore the function of an organ. This seems to be true for the lung as well. However, understanding of the endogenous stem/progenitor cell population involved and the underlying mechanisms that control the proper regeneration of lung tissue remains broadly elusive. Although several animal injury models provide evidence of compartmental stem/progenitor activation and proliferation, the underlying signalling pathway that initiates this regenerative activity is largely unknown. Evidence suggests that, in many disease conditions, prenatal lung developmental signalling is reactivated. Our understanding in mammalian lung development, in particular the process of alveologenesis, is poorly understood. However, the recent advent of organotypic *ex vivo* models, such as “tracheosphere” and “alveolosphere” culture offer novel platforms for studying stem/progenitor dynamics and associated signalling pathways that could be involved in lung regeneration. Furthermore, derivation of lung progenitors from ESCs and iPSCs for the repair of lung after injury and disease condition opens another exciting avenue towards development of regenerative therapeutics for lung disease. In this case, however, the pluripotent nature of ESCs or iPSCs could present a potential risk of teratogenic effects, which needs to be rigorously addressed before moving into human clinical trial. Much study, so far, has been done to evaluate MSC-mediated cell therapy in various lung conditions, albeit mostly in animal models. In this case, it is important to note that the bulk of studies suggest the infused MSCs exhibits reparative/healing effects mostly through paracrine or immunomodulatory effects on recipient lung tissue, but not by engraftment. Thus, it is imperative to view MSC therapy as cell-based immunomodulatory therapy rather than as attempts to regenerate or reconstitute lung tissues [6]. Nonetheless, much effort has been taken to date to understand the molecular mechanisms of lung development, disease dynamics and its regenerative process. Much, of course, still remains to be determined. By combining the strength of basic studies along with rational clinical approaches we can fully unravel the power and promise of next-generation regenerative therapeutics in the field of lung disease in the coming years.
